# Brazilian Nutritional Consensus in Hematopoietic Stem Cell Transplantation: Elderly

**DOI:** 10.31744/einstein_journal/2019AE4340

**Published:** 2019-05-14

**Authors:** Sandra Elisa Adami Batista Gonçalves, Andreza Alice Feitosa Ribeiro, Erika Yuri Hirose, Fabio Pires de Souza Santos, Flávia Munhoz Ferreira, Ludmila de Oliveira Muniz Koch, Márcia Tanaka, Mayara Santos de Souza, Polianna Mara Rodrigues Souza, Thiago Jose Martins Gonçalves, Andrea Z Pereira

**Affiliations:** 1 Hospital Israelita Albert Einstein, São Paulo, SP, Brazil.; 2 Centro Prevent Senior, São Paulo, SP, Brazil.; 3 Centro de Transplante de Medula Óssea, Rio de Janeiro, RJ, Brazil.; 4 Hospital Sírio-Libanês, São Paulo, SP, Brazil.

**Keywords:** Hematopoietic stem cell transplantation, Aged, Nutritional status, Nutrition assessment, Transplante de células-tronco hematopoéticas, Idosos, Estado nutricional, Avaliação nutricional

## Abstract

The Brazilian Nutritional Consensus in Hematopoietic Stem Cell Transplantation: Elderly was elaborated by nutritionists, nutrologists and hematologists physicians from 15 Brazilians reference centers in hematopoietic stem cell transplantation, in order to emphasize the importancy of nutritional status and the body composition during the treatment, as well as the main characteristics related to patient’s nutritional assessment. Establishing the consensus, we intended to improve and standardize the nutritional therapy during the hematopoietic stem cell transplantation. The Consensus was approved by the Brazilian Society of Bone Marrow Transplantation.

## RESUMO

O Consenso Brasileiro de Nutrição em Transplante de Células-Tronco Hematopoiéticas: Idoso foi elaborado com a participação de nutricionistas, médicos nutrólogos e médicos hematologistas de 15 centros brasileiros referência em transplante de células-tronco hematopoiéticas, com o objetivo de salientar a importância do estado nutricional e da composição corporal durante o tratamento, bem como as principais características relacionadas à avaliação nutricional do paciente. As intenções, ao se estabelecer o consenso, foram aprimorar e padronizar a terapia nutricional durante o transplante de células-tronco hematopoiéticas. O consenso foi aprovado pela Sociedade Brasileira de Transplante de Médula Óssea.

## INTRODUCTION

Population aging is a worldwide phenomenon, which has for long brought concerns regarding its socioeconomic consequences, many of which are related to changes in the epidemiological profile of the population, and is considered the main social transformation of the 21^st^ century.^(^ ^1^ ^,^ ^2^ ^)^ According to data from the United Nations (UN), life expectancy at birth is currently above 80 years in 33 countries, whereas 5 years ago only 19 countries had reached this mark.^(^ ^1^ ^)^ The association of lower fertility and mortality rates and increased longevity has resulted in population aging. The drop in fertility rates made younger age groups less representative in the total population. Lower mortality rates and increased life expectancy also contributed to this process.^(^ ^1^ ^,^ ^2^ ^)^

In 1950, there were 205 million people aged 60 years and over in the world, according to the United Nations (UN). In 2000, they were approximately 400 million and, in 2015, more than 900 million elderly individuals. According to estimates, by 2030, this number will increase by 56%, rising to 1.4 billion, and in 2050, it will reach more than 2 billion people. It is worth noting the proportion of the elderly population that has most increased is the so-called “old-old or long-living elderly”, *i.e*., people aged 80 years or older. The same estimates indicate that, in 2050, they will be 434 million, or more than three times the number in 2015, which was 125 million in total.^(^ ^1^ ^,^ ^2^ ^)^

Brazil is also at full speed in this demographic transition. While in the 1960’s, there were about 3 million people aged 60 years and over in the country, in 2000 they already totaled more than 14 million people. In the 1991 demographic census, the elderly accounted for 7.3% of Brazilian population; in 2000, 8.6%; in 2006, 10.2% and, in 2015, 14.3%.^(^ ^3^ ^-^ ^5^ ^)^ Estimates of the Brazilian Institute of Geography and Statistics (IBGE) indicate that, in 2020, the elderly account for 15% of Brazilian population, rising to 18% in 2050, or approximately 38 million people. Furthermore, in 2060, we will have 19 million seniors aged 80 years and older. Brazil will be the sixth country with the highest number of elderly individuals.^(^ ^3^ ^-^ ^5^ ^)^ The World Bank estimates that, in the next 40 years, the Brazilian elderly population will grow at a rate of 3.2% per year, while the total population will grow at a rate of 0.3%.^(^ ^4^ ^)^

This new demographic scenario brings significant challenges for the health sector. As the population ages, the prevalence of chronic degenerative diseases increases along with their consequences. These include malignant neoplasms, both solid tumors and hematological malignancies.

Data from the *Instituto Nacional do Câncer José Alencar Gomes da Silva* (INCA) show that, with the increase in population aging in recent decades, there was also an exponential increase in the incidence of cancer in the Brazilian elderly population,^(^ ^6^ ^)^ and this is a major challenge faced by oncologists, hematologist-oncologists and geriatricians.^(^ ^7^ ^,^ ^8^ ^)^ Approximately 70% of cancer cases are diagnosed in individuals aged 60 years and over, and cancer is the cause of 70% of deaths in this age range.^(^ ^6^ ^,^ ^9^ ^)^ The INCA estimates indicated that approximately 600 thousand new cases of cancer would occur in Brazil, in the period 2016-2017. Excluding non-melanoma skin cancer, which accounts for approximately 180 thousand new cases, there should be approximately 420 thousand new cancer cases.^(^ ^6^ ^)^

With regards to hematological malignancies, it was estimated that approximately 5,210 new cases of non-Hodgkin’s lymphoma (NHL) in men and 5,030 in women would occur in Brazil in 2016, corresponding to an estimated risk of 5.27 new cases per 100 thousand men, and 4.88 per 100 thousand women. As for Hodgkin’s lymphoma (LH), it was estimated that 1,460 new cases would occur in men and 1,010 in women, corresponding to an estimated risk of 1.46 new case per 100 thousand men and 0.93 per 100 thousand women.^(^ ^6^ ^)^

Considering leukemias, 5,540 new cases were expected in men and 4,530 in women. These values corresponded to an estimated risk of 5.63 new cases per 100 thousand men and 4.38 per 100 thousand women.^(^ ^6^ ^)^

There are no detailed estimates for multiple myeloma in the latest INCA publication about cancer incidence in Brazil.

**Figure 1:**
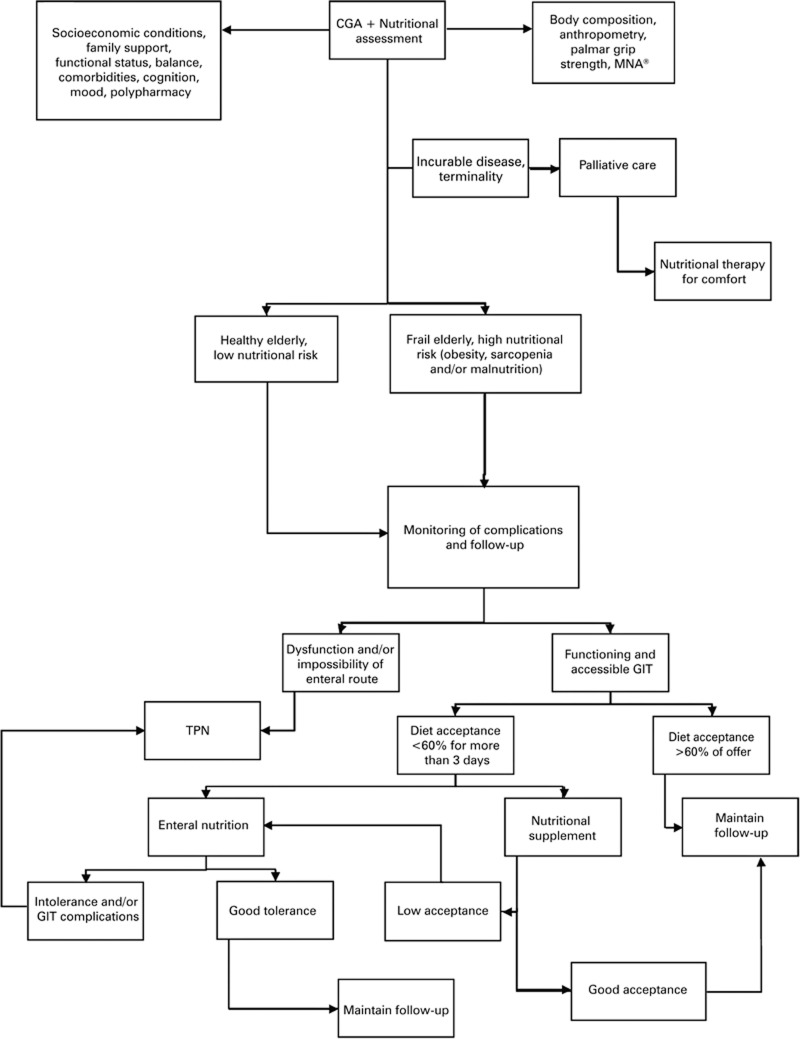
Practical organization chart of nutritional approaches to be applied

CGA: comprehensive geriatric assessment; MNA^®^: Mini-Nutritional Assessment^®^; TPN: total parenteral nutrition; GIT: gastrointestinal tract.

Figure 1 provides a brief outline of the nutritional approaches recommended.

## HEMATOPOIETIC STEM CELL TRANSPLANTATION IN THE ELDERLY

Hematopoietic stem cell transplantation (HSCT) is a potentially curative treatment which can prolong survival for many patients diagnosed with hematological malignancies. However, due to its high related morbidity, for a long time, this treatment strategy was restricted to young patients, since aging is related to higher rates of comorbidities and functional impairment, and a consequent increase in the risk of toxicity and adverse events.^(^ ^10^ ^,^ ^11^ ^)^

However, today we known that the aging process is not the same for all individuals. It is a heterogeneous process, influenced by genetic and environmental factors, which makes chronological age alone not the best variable to be considered in decision-making regarding therapies.^(^ ^10^ ^-^ ^13^ ^)^

The number of patients eligible for HSCT has increased considerably, probably due to increased life expectancy associated with earlier diagnosis and greater access to health services. With the emergence of the less toxic, so-called non-myeloablative conditioning regimens, and the recognition of the importance of supportive therapies, elderly patients are increasingly becoming candidates for HSCT. According to data from the Center for International Blood and Marrow Transplant Research (CIBMTR), in recent years there has been a significant increase in the number of elderly patients subjected to autologous and allogeneic HSCT. Whereas between 1994 and 1995 less than 1% of patients undergoing autologous HSCT were aged 70 years and over, between 2004 and 2005, they already accounted for 5% of total.^(^ ^10^ ^,^ ^11^ ^)^ In this same period, the percentage of autologous HSCT in patients aged between 60 and 69 years increased from 6% to 25% of total. As for allogeneic HSCT, the number of patients aged 60 years and over undergoing the procedure increased 13-fold, between 1994 and 2005.^(^ ^10^ ^,^ ^11^ ^)^

Hematological malignancies are more common in older people, and many have a higher incidence in the elderly (particularly aged between 60 and 70 years), such as acute myeloid leukemia (AML), myelodysplastic syndromes (MDS), multiple myeloma and NHL. For various reasons, the elderly have a worse prognosis than younger patients, either due to a higher incidence of comorbidities or biological aspects of the disease at this stage of life, which lead to a poorer progression.^(^ ^14^ ^)^ In the elderly, *e.g.*, there is a higher incidence of expression of genes such as MDR-1, as well as microsatellite instability, in malignant cells, providing them, respectively, with greater resistance to chemotherapeutic agents,^(^ ^15^ ^)^ and poorer prognosis in acute leukemias.^(^ ^16^ ^,^ ^17^ ^)^ The overall one-year survival of elderly patients with high-risk AML/MDS is less than 30%, and the prognosis of aggressive NHL recurring after autologous HSCT is very unfavorable.^(^ ^18^ ^,^ ^19^ ^)^

Hematopoietic stem cell transplantation may lead to cure or prolonged remission in many malignant or non-malignant diseases. Until two decades ago, its application was restricted by a maximum age limit, which was, usually, 65 years for autologous and 55 years for allogeneic transplantation. Fortunately, advances in HSCT have enabled lower toxicity in conditioning regimens, shorter duration of neutropenia, lower treatment-related mortality rates, and greater focus on immunomodulation and targeted therapies for maintenance of remission in hematological malignancies.^(^ ^14^ ^)^

In the last decade, there is increasingly more data on the safety and efficacy of this procedure in the elderly.^(^ ^20^ ^)^ Patient age is no longer considered an impediment for HSCT, and greater focus is now placed on more relevant assessments of comorbidity and functionality indices that reflect the physiological rather than chronological age.^(^ ^14^ ^,^ ^21^ ^)^ Sorror et al.,^(^ ^22^ ^)^ showed better expectancies in their prospective study with 372 patients aged 60 and 75 with high-risk hematological malignancies, undergoing non-myeloablative, allogeneic HSCT, and demonstrated a one-year treatment-related mortality rate of 20% (95% confidence interval - 95%CI: 22%-32%), 65% of graft *versus* host disease (GVHD) and 33% recurrence.^(^ ^22^ ^)^

Center for International Blood and Marrow Transplant Research reports show that the number of autologous transplsxantations in elderly patients continues to grow. Approximately 50% of autologous transplants for multiple myeloma and lymphoma performed in 2015 were in patients aged over 60 years, and 12% of total autologous transplants were in patients aged over 70 years. The number of allogeneic transplants also continues to grow, given that 30% of all allogeneic transplants performed in 2015 were in patients over 60 years, and patients over 70 years accounted for 4.4% of total. This increase is due to improved supportive measures, such as better monitoring and treatment of infections; in the case of allogeneic transplants, this is also due to the introduction of reduced intensity, or non-myeloablative conditioning regimens, and the new immunosuppressive regimens.

Hematopoietic stem cell transplantation with reduced intensity and/or non-myeloablative conditioning decreases the acute toxicity of transplantation, allowing for longer disease-free survival in elderly patients, even those over the age of 70 years. A study by Brunner et al.,^(^ ^20^ ^)^ with 54 patients aged over 70 years undergoing HSCT for hematological malignancies showed that non-HSCT-related mortality was approximately 3.7% by day +100, and approximately 5.6% by 2 years after the transplant, resulting in a 2-year disease-free survival of 39%.

Another important aspect of allogeneic HSCT in elderly patients is linked with the donor. Most donors are relatives of the same age, with a comorbidity profile similar to that of elderly patients candidate for allogeneic HSCT, which limits the possibility of donation.^(^ ^21^ ^)^ Some strategies can be used to obtain a suitable product, and several transplant teams have proposed and enabled unrelated allogeneic HSCT or alternative sources, such as haploidentical HSCT. Unfortunately there are few randomized or prospective studies with elderly patients undergoing HSCT.

Finally, an important aspect is the increasing use of comorbidity indices for the pre-transplant evaluation, which allows to predict the overall survival of patients and transplant-related mortality. These indices, along the functional assessment, such as the Karnofsky Performance Status (KPS), are important instruments in the initial evaluation and may even contraindicate HSCT.^(^ ^23^ ^)^ However, most studies showed that these two indices are insufficient, and tools to provide a more accurate prognosis are needed. One of them is the Comprehensive Geriatric Assessment (CGA), which proved to be an independent prognostic factor for overall survival of elderly patients.^(^ ^24^ ^)^

The number of elderly candidates for HSCT tends to continue to grow, considering that the population is aging and the number of elderly patients diagnosed with hematological malignancies is increasing. Thus, it is important to define which elderly patients can actually tolerate the procedure and which would benefit from a less intensive treatment, and furthermore, what are the main variables that could predict better or poorer outcomes and support this decision.

## IMPORTANCE OF NUTRITIONAL STATUS IN HEMATOPOIETIC STEM CELL TRANSPLANTATION IN THE ELDERLY

Although aging is not necessarily linked to diseases and disabilities, several senescence-related conditions in the physical, cognitive and social spheres, contribute to the elderly’s greater susceptibility to adverse health events, increasing the risk of malnutrition. Dementia, depression, chronic renal diseases, cardiac and pulmonary diseases, oral and dental disorders, and dependence on others for basic activities of daily living, such as eating, deteriorate the clinical condition of the elderly. Thus, patients aged over 70 years often become malnourished and, at this age range, the intake of proteins and other nutrients is usually inadequate.^(^ ^25^ ^-^ ^27^ ^)^

Regarding adipose tissue, it progressively increases during adulthood, similarly in both genders, until the seventh decade of life;^(^ ^28^ ^)^ from then on, there is a predominant increase in visceral fat, combined with a decrease in subcutaneous fat, which may occur independently of changes in body weight, total adiposity or waist circumference.^(^ ^29^ ^)^ Muscle mass represents 40% of body weight in adults and 30% in the elderly. The decrease in muscle mass is correlated with less strength and, after 60 years of age, it can decrease up to 3% per year.^(^ ^30^ ^)^

As we age, weight usually increases, due to increased body fat and loss of muscle mass.^(^ ^31^ ^,^ ^32^ ^)^ International data show that 5% to 13% of individuals ≥60 years of age have low muscle mass, and this prevalence increases to up to 50% in those aged ≥80 years.^(^ ^33^ ^)^ The few studies available in the Brazilian population do not provide consistent data in terms of findings and methods.^(^ ^34^ ^,^ ^35^ ^)^

The elderly are at higher risk for chemotherapy-induced toxicity, compared to young adults - a fact attributed to the decrease in muscle mass.^(^ ^36^ ^,^ ^37^ ^)^ In addition, they are less likely to receive chemotherapy, due to concerns about their ability to withstand the treatment.^(^ ^38^ ^)^ Therefore, in order to decide on the best treatment for each patient and avoid serious adverse effects, it is important to identify the elderly who are at nutritional risk and risk of poorer functional status.^(^ ^38^ ^)^

Although most patients are not malnourished when they start the HSCT, patients with low weight, obese and those presenting with worsening of nutritional status during the procedure have a high risk of early death after HSCT, since these are negative prognostic factors.^(^ ^39^ ^)^

Both protein-calorie malnutrition and obesity increase the morbidity and mortality risk, length of hospital stay, duration of immunosuppressant use, and the chance of developing GVHD.^(^ ^40^ ^,^ ^41^ ^)^

Obesity, with a prevalence in HSCT varying between 10% and 34%, is associated with a higher incidence of GVHD, infections and mortality.^(^ ^42^ ^)^ A recent study performed in patients undergoing allogeneic HSCT showed an inverse association between areas of visceral and peripheral fat and the disease-free period.^(^ ^43^ ^)^

In HSCT, the decrease in muscle strength and mass is associated, among other factors, with the use of corticosteroids and immunosuppressant agents, leading to poorer prognosis.^(^ ^44^ ^,^ ^45^ ^)^ In allogeneic HSCT, this decrease is associated with a higher prevalence of chronic GVHD and low physical performance.^(^ ^44^ ^,^ ^45^ ^)^

In the elderly, there is an increased prevalence of sarcopenia,^(^ ^46^ ^)^ defined by loss of muscle mass associated with decreased muscle strength, and this is not only associated with a worse prognosis for different clinical entities,^(^ ^47^ ^-^ ^51^ ^)^ but is also a negative prognostic factor for hematological malignancies.^(^ ^52^ ^)^

Although there is a tendency to associate sarcopenia and malnutrition, the first also occurs in obese patients and is called sarcopenic obesity.^(^ ^53^ ^,^ ^54^ ^)^ It is difficult to diagnose, because it depends on a method to assess body composition, in addition to having a complex etiology, involving life style, as well as endocrine, vascular and immunological factors.^(^ ^53^ ^,^ ^54^ ^)^ In cancer patients, sarcopenic obesity reduces survival.^(^ ^55^ ^)^

Several factors influence patients’ clinical outcomes, such as the type and stage of the disease, conditioning regimen, type of HSCT (autologous, allogeneic or haploidentical), source of stem cells (peripheral blood, bone marrow or umbilical cord), age and nutritional status.^(^ ^56^ ^)^

The nutritional assessment of elderly patients before and during HSCT could identify those requiring early nutritional intervention, prevent complications and, consequently, reduce the length of stay and the need for intensive care, increase survival, and improve the quality of life and clinical care provided to the patient, thus influencing the clinical and nutritional outcomes of the disease.^(^ ^56^ ^)^

## GERIATRIC ASSESSMENT IN HEMATOPOIETIC STEM CELL TRANSPLANTATION

### Pre-hematopoietic stem cell transplantation assessment of elderly patients

In the standard pre-HSCT assessment, some traditional tools are routinely used to establish the transplantation prognosis, including assessments of disease status, type of donor, cell source and, performance status, which can be evaluated by both the Karnofsky Performance Status (KPS) and the Eastern Cooperative Oncology Group (ECOG) score. The assessment also includes comorbidity indices and prognostic indices relative to survival.^(^ ^57^ ^)^ The development of a specific comorbidity index for hematopoietic cell transplantation by Sorror et al., the Hematopoietic Cell Transplantation-Specific Comorbidity Index (HCT-CI), led to important progress in how the post-transplant prognosis is predicted. It is a comorbidity scoring system that predicts transplantation-related toxicity and overall survival.^(^ ^58^ ^)^

Nevertheless, although quite comprehensive, the standard assessment alone proved insufficient to evaluate all relevant dimensions of health conditions affecting elderly patients. Aging is a very heterogeneous process, and the chronological age assessment alone does not predict the real conditions of elderly patients in terms of functional and cognitive capacities; nutritional status; comorbidity profile and polypharmacy use; emotional state; social support, and presence of geriatric syndromes. Thus, adequate attention should be paid to the physiological and psychosocial changes resulting from the aging process, to allow for detection of previously unknown or underdiagnosed problems that may interfere with the safety and efficacy of cancer treatment.^(^ ^9^ ^-^ ^13^ ^)^

A more comprehensive and multidimensional health assessment of the elderly can be obtained using the CGA, which can contribute to the careful selection of elderly candidates for HSCT.^(^ ^11^ ^)^

The CGA is an instrument supported by the medical literature to aid in determining deficiencies, disabilities or handicaps, establishing the needs and goals of patient care and planning for long-term follow-up.^(^ ^59^ ^)^ It is a multidimensional assessment of the elderly, which considers multiple dimensions using instruments that assess functionality; balance, gait and mobility conditions; cognitive function; sensory changes; emotional changes and conditions; socioeconomic conditions, availability and suitability of family and social support; environmental conditions; nutritional status and risk; presence of comorbidities, geriatric syndromes and polypharmacy; and drug-drug interaction profile.^(^ ^7^ ^-^ ^9^ ^,^ ^12^ ^,^ ^13^ ^,^ ^59^ ^)^

The functional status of the elderly can be evaluated using specific functional scales that have been validated for the geriatric population, such as the Katz Scale for Basic Activities of Daily Living, and the Lawton Scale for Instrumental Activities of Daily Living. Balance and mobility can be evaluated by instruments such as the Timed Up and Go (TUG) test, and cognitive capacity by instruments such as the Mini Mental State Examination (MMSE), as well as the clock-drawing and verbal fluency tests, considered as cognitive screening tests in this setting. To evaluate mood changes, it is possible to use the Geriatric Depression Scale (GDS) adapted by Yesavage. Medical conditions are evaluated based on a list of comorbidities, as well as comorbidity indices, such as the Charlson Comorbidity Index and/or the Cumulative Illness Rating Scale for Geriatric (CIRS-G); drug list and assessment of polypharmacy use and drug-drug interactions; and presence of sensory deficiencies and geriatric syndromes, such as falls, *e.g*. The nutritional assessment can be through anthropometric measurements, measurement of palmar grip strength using a suitable hydraulic dynamometer, and implementation of the Mini-Nutritional Assessment^®^ (MNA^®^). Social functioning can be assessed by questionnaires addressing socioeconomic and environmental conditions, as well as the availability and suitability of family support and additional support networks.^(^ ^59^ ^-^ ^64^ ^)^ Also, one can apply chemotherapy-induced toxicity scores, such as the Chemotherapy Risk Assessment Scale for High-Age Patients Score (CRASH) and Hurria’s toxicity score.^(^ ^65^ ^,^ ^66^ ^)^

Through this comprehensive evaluation, it is possible to identify potential vulnerabilities and/or fragilities that may expose elderly patients to a higher risk of toxicities and complications, compromising their treatment. By identifying these factors, a personalized therapeutic plan can be established, creating not only the possibility of offering appropriate cancer treatment, but also specific measures to solve any problems encountered, aiming to maintain independence and quality of life. The assessment assists in the identification of more frail elderly patients, at high risk for unfavorable outcomes, for whom it is necessary to judge the real benefits of HSCT much more judiciously, and consider the possibility of referring them for supportive therapies and palliative care.

Elderly patients are considered frail when they meet the requirements for a diagnosis of frailty syndrome according to Fried’s frailty criteria, *i.e.*, presence of three or more of the following: reduced speed, reduced strength, reduced physical activity, exhaustion and weight loss ≥5% in the last year; or two of the following: age over 85 years, more than two disabilities, multiple comorbidities, or presence of geriatric syndrome. These elderly patients, who are frequently not identified as such in a usual clinical evaluation, are at high risk for negative outcomes, such as higher chemotherapy-induced toxicity, irreversible functional decline and death.^(^ ^11^ ^)^ At least two important studies have shown that, despite being considered as having excellent functional status in the usual pre-HSCT assessment, important functional *deficits* and geriatric syndromes were identified in elderly patients previously eligible for HSCT, after they were subjected to the CGA. Approximately one quarter of these met the criteria for diagnosis of frailty syndrome.^(^ ^11^ ^,^ ^67^ ^)^

The elderly patients considered healthy are those with no geriatric syndromes, totally independent, and whose comorbidities are controlled with no clinical repercussions. Patients who do not meet the criteria for frailty syndrome, but rather for a pre-frail state, with no other diagnosed geriatric syndromes and having a maximum of two uncontrolled comorbidities and some dependence for Instrumental Activities of Daily Living, but total independence for Basic Activities of Daily Living, are considered vulnerable.^(^ ^59^ ^-^ ^64^ ^)^ Although the CGA can be useful in the preparation of rehabilitation measures so that vulnerable elderly patients can benefit from the best cancer treatment with the least toxicity risk, in case of hematological malignancies, not always will it be possible to use the CGA data to promote targeted interventions for reversible conditions before HSCT, because time is valuable in the treatment of hematological malignancies, and delays in HSCT after its indication can lead to worse outcomes and prognoses.^(^ ^11^ ^)^

The implementation of a geriatric assessment can, without a doubt, be useful for proper selection of elderly candidates for HSCT, since it can identify frail elderly patients at higher risk for poor outcomes; however, further studies are needed in order to define the main variables associated with a better or worse prognosis, which could contribute for better selection of candidates for the procedure, particularly when it comes to those considered vulnerable.

## MONITORING DURING AND AFTER HEMATOPOIETIC STEM CELL TRANSPLANTATION

Despite studies pointing to the CGA as the main ancillary instrument for selecting elderly candidates for HSCT, one of its main roles continues to be establishing a care plan focusing on preventing functional loss and improving quality of life, since it allows for early detection of deficiencies and handicaps. Thus, the CGA can be applied whenever appropriate, aiming to readjust the initial plan when necessary.

The elderly are particularly susceptible to poor outcomes associated with hospitalization, especially when there is prolonged bed rest, which can cause accelerated loss of bone and muscle mass, which worsens their functional capacity, often irreversibly.^(^ ^68^ ^,^ ^69^ ^)^ Maintaining nutritional status, in this situation, is especially important, given that its deterioration can bring serious consequences. This happens because, over the course of the HSCT, there is usually an unfavorable scenario, with simultaneous presence of decreased food intake and absorptive changes associated with the toxicity of cytoreductive therapy (which causes nausea, vomiting, loss of appetite and dysgeusia) and increased metabolic requirements.^(^ ^70^ ^)^

Still focusing on prevention of functional loss and other complications, it is of utmost importance to properly manage toxicities and symptoms related to the HSCT process, which may be present since the conditioning stage until a few weeks after the procedure. The main symptoms include fatigue, nausea and vomiting, diarrhea, pain, mucositis and dyspnea. It is worth noting that uncontrolled symptoms decrease compliance to rehabilitation measures.

Another important point is the strict control of comorbidities, because, even if previously controlled, they may decompensate during the transplantation, particularly cardiovascular diseases, which may manifest as heart failure and arrhythmias.^(^ ^10^ ^)^

### Nutritional assessment and intervention before, during and after hematopoietic stem cell transplantation

Elderly patients with prior nutritional risk undergoing HSCT routinely require individualized and optimized nutritional therapy (NT), which must be immediately started since the pre-HSCT stage, especially in the presence of malnutrition.

Nutritional support during HSCT aims to maintain or improve nutritional status, and provide substrate for recovery of the hematopoietic and immune systems, as well as minimizing consequences of the conditioning regimen and helping maintain immunocompetence.^(^ ^71^ ^-^ ^73^ ^)^

The guidelines do not differentiate between NT in adults and the elderly. Generally, the first form of nutritional support should be patient counseling to help manage the adverse effects of the treatment, and adjust patients’ diets with better tolerated foods.^(^ ^74^ ^)^

Nutritional therapy in HSCT aims to^(^ ^75^ ^)^ maintain and/or recover the nutritional status post-HSCT; avoid or minimize nutritional deficiencies resulting from chemotherapy and/or radiotherapy; keep the gastrointestinal tract active; prevent protein-calorie malnutrition; and promote better oral intake, providing adequate substrate for recovery of the hematopoietic and immune systems.

Usually, malnutrition is caused by conditioning regimens with high-dose chemotherapy and/or radiation therapy, leading to effects such as nausea, vomiting, diarrhea and mucositis. These serious gastrointestinal toxicities usually disturb oral feeding, increasing the risk of malnutrition and life-threatening infection.

These complications affect oral feeding and drinking and, together with development of the systemic inflammatory response syndrome, may lead to anorexia and cachexia. Thus, the nutritional assessment in elderly patients undergoing HSCT is very important and can help in the decision-making process for nutritional support.^(^ ^76^ ^)^

To investigate the nutritional profile of these elderly patients before and after HSCT and explore the optimum methods to assess their nutritional status, we have four major nutritional screening tools, including the Nutrition Risk Screening 2002 (NRS 2002), MNA^®^, Subjective Global Assessment (SGA) and Malnutrition Universal Screening Tools (MUST) - which, in combination with anthropometry, allow for an extensive examination and evaluation of the risks and nutritional status of elderly patients undergoing HSCT.^(^ ^77^ ^,^ ^78^ ^)^ In the elderly, the most commonly used is the MNA^®^.

Nutritional assessment, which starts in the pre-HSCT period, is the first step for detection and treatment of malnutrition. Patients undergoing HSCT are considered to be at increased risk for malnutrition before and after HSCT, and an abnormal nutritional status before HSCT is a negative prognostic factor for these patients, interfering with the grafting time.^(^ ^79^ ^)^

Due to limitations of the current nutritional assessment methods, it is very important to adopt practical, low cost methods, with minimal manipulation of patients undergoing HSCT.

Hematopoietic stem cell transplantation in the elderly is relatively recent, and currently there are no specific instruments for nutritional assessment. Most centers use the nutritional section of the CGA and the standard evaluations for adults. No nutritional, anthropometric or biochemical assessment method is free from faults and/or contraindications. Each center should research and identify the most adequate method for their reality, avoiding excessive manipulation of these patients. We suggest the use of a method to measure body mass, skin folds or bioimpedance, with longer intervals - biweekly -, since fewer evaluations do not affect patient evolution.

## ASSESSMENT OF BODY COMPOSITION IN ELDERLY PATIENTS UNDERGOING HEMATOPOIETIC STEM CELL TRANSPLANTATION

In HSCT, body composition has been studied, and important correlations have been found with complications and survival. However, there are still few studies about the population of elderly patients undergoing HSCT.^(^ ^80^ ^-^ ^82^ ^)^

Allogeneic HSCT causes most body composition changes in adults, but, in the elderly, even autologous transplantation can be associated with loss of muscle mass, especially when the patient had previous muscle depletion at the beginning of the procedure, which shows that the elderly population undergoing HSCT has peculiarities regarding body composition.^(^ ^83^ ^)^

The analysis of computed tomography (CT) images taken in specific views – of the third lumbar vertebra and the fourth thoracic vertebra –, shows good correlation with fat mass and lean muscle mass throughout the body.^(^ ^84^ ^-^ ^87^ ^)^ This method also allows for assessment of the muscle radiodensity, which is defined as the mean radiation attenuation in Hounsfield units. Low muscle radiodensity in some studies seems to be a better prognostic indicator than sarcopenia in solid tumors and hematological malignancies.^(^ ^88^ ^,^ ^89^ ^)^ Since most HSCT patients have it assessed in the pre-HSCT evaluation, this method can be used, even though it depends on the availability of a specific software program, which limits its use in all services.^(^ ^90^ ^,^ ^91^ ^)^ In elderly patients, CT allows for an estimation of the presence of sarcopenia, in addition to the body composition itself.^(^ ^55^ ^,^ ^90^ ^-^ ^92^ ^)^

Another method that enables assessment of sarcopenia, which is more practical and low cost when compared to CT, is ultrasound (US).^(^ ^93^ ^)^ Some studies use the quadriceps femoris thickness and echogenicity, which reflects the amount of muscle fibers, to assess sarcopenia in elderly patients.^(^ ^50^ ^,^ ^94^ ^-^ ^96^ ^)^ In addition, US enables evaluation of visceral fat, which is a prognostic factor in HSCT.^(^ ^97^ ^-^ ^99^ ^)^

Unfortunately, this is not yet a regular practice in Brazilian HSCT services; nonetheless, in elderly patients, it can enhance NT at all stages of HSCT, improving patient prognosis. Among the methods for assessment of body composition, the service must choose the one that is more practical and has the best cost-benefit ratio for patients. The most important is to turn this evaluation into a regular tool of the nutritional and geriatric assessment.

## ORAL, PARENTERAL AND ENTERAL NUTRITION

### Oral nutrition

The feeding route must be chosen in accordance with clinical symptoms, digestive disorders and the adequacy of oral intake, and more than one route may be used simultaneously to meet the nutritional needs of the patient.^(^ ^100^ ^,^ ^101^ ^)^

The neutropenia associated with the conditioning therapy leads to the indication of low-microbial diets, which restrict foods associated with the risk of infection, such as eggs and raw or incompletely cooked meats, non-pasteurized dairy products (milk, cheese, butter, yogurt), raw fruit and its byproducts, fresh and boiled vegetables, raw oily fruit, and unboiled tap water.^(^ ^100^ ^)^

Despite the lack of knowledge about the effects of a low-microbial diet, it is also recommended to provide dietary counseling regarding foods which are safe to eat during this period, as well as the correct cooking and washing techniques to prevent infections.^(^ ^100^ ^)^

Patients who are malnourished or at risk of malnutrition, and those with poor food acceptance (≤75% acceptance of nutritional requirements for more than 3 days) are candidates to initiate adjustment of their calorie target with supplements, preferably of the high-calorie and high-protein types. These measures may provide malnourished elderly patients with better maintenance or recovery of their body weight, and decreased mortality, with subsequent decrease of hospital care costs.^(^ ^71^ ^,^ ^72^ ^)^

Currently, there is a great variety of supplements that are palatable, easy to handle and to consume, and suitable for all age ranges.^(^ ^102^ ^)^ There is a concern regarding compliance to the quantity and type of supplements prescribed to reach nutritional goals and maximize the clinical efficacy and cost-effectiveness, as well as avoid waste.^(^ ^71^ ^,^ ^102^ ^,^ ^103^ ^)^ Adherence to supplements should be monitored, as well as patient tolerance, to allow for new adjustments when needed.^(^ ^104^ ^)^ In case of intolerance, higher-density and energy-content supplements can be used to reduce the volume ingested.^(^ ^105^ ^)^

A strategy to improve compliance to nutritional supplements is demonstrated in the program called MedPass Nutrition, in which nurses offer around 50 to 60mL of liquid supplements throughout the patient’s daily drug routine. In this case, the supplement is presented to the patient as part of their oral drugs rather than a snack or part of a meal, ensuring that the patient receives nutritional support along with their medication. The patient adherence analysis in this program was positive: patients accepted 95.8% of total supplement volume prescribed in the first 4 weeks, and 86.6% of volume in the last 4 weeks of the study. This resulted in major improvement of their weight gain and body mass index (BMI).^(^ ^103^ ^,^ ^105^ ^)^

### Enteral nutrition

During HSCT, nutritional support must be routinely offered to prevent malnutrition secondary to gastrointestinal toxicity induced by the conditioning regimen, or to perform the necessary adjustments to the increased demand of a catabolic state.^(^ ^106^ ^,^ ^107^ ^)^

When oral NT is inadequate, be it due to the impossibility of using the digestive tract or an intake lower than 60% of nutritional needs for up to 5 consecutive days, with no expected improvement, in patients who maintain total or partial functionality of the gastrointestinal tract, enteral nutritional therapy (ENT) must be the treatment of choice.^(^ ^74^ ^,^ ^108^ ^)^

Regarding the positioning of the tube, a nasogastric (NGT) or nasoenteral (NET) tube can be used, which must be chosen according to the signs and symptoms presented by the patient.^(^ ^103^ ^)^ Studies indicate that the insertion of a NGT, in the same week of the hematopoietic cell infusion, improves tolerance of enteral nutrition during the treatment.^(^ ^103^ ^,^ ^109^ ^,^ ^110^ ^)^ However, the great challenge is to secure safe enteral access after the transplantation, due to coagulation disorders, risk of aspiration pneumonia, diarrhea, ileus, abdominal pain, vomiting and gastroparesis.^(^ ^111^ ^)^ The multidisciplinary team must monitor, on a daily basis, all clinical and laboratory data, signs, symptoms, physical and functional exams, as well as their accurate charting.^(^ ^71^ ^)^

To determine the best formula to be used, the individual characteristics of each clinical case must be considered. The use of low-osmolarity, polymeric enteral formulas, with continuous infusion and progressive increase of the volumes offered, is generally well tolerated.^(^ ^110^ ^)^

Enteral nutritional therapy is always preferable due to it being the most physiological, preserving the integrity of the intestinal mucosa, and ensuring the lowest bacterial translocation rates, in addition to favoring good glycemic control and lower incidence of infection-related complications.^(^ ^72^ ^,^ ^111^ ^,^ ^112^ ^)^

However, TPN is still the first choice of HSCT teams due to the digestive toxicity caused by high-dose chemotherapy used for immune ablation during the pre-HSCT period, which hinders the insertion of the NGT or NET.^(^ ^110^ ^)^

### Parenteral nutrition

Parenteral nutrition is an alternative way of feeding patients who cannot have their nutritional needs met by the enteral or oral route.^(^ ^113^ ^)^ In patients undergoing HSCT, digestive dysfunction starts as early as in the pre-transplant phase, as a result of an aggressive, myeloablative conditioning period, which can last 3 to 4 weeks during immunosuppressive therapy.^(^ ^114^ ^,^ ^115^ ^)^ In this period, various digestive changes occur, such as nausea, vomiting, mucositis, diarrhea, and nutrient malabsorption; consequently, oral and enteral feeding are impaired, and the nutritional status of the patient deteriorates.^(^ ^4^ ^)^ At the same time, during this period, the energy requirements can increase to up to three times the basal metabolic rate, making it even more difficult to adjust the intake of nutrients to such a high demand.^(^ ^116^ ^)^

The literature is contradictory about the real benefits of TPN in the context of HSCT. Studies show that its prophylactic use could be associated with major adverse effects and, therefore, the risk-benefit ratio must be considered.^(^ ^56^ ^)^ Szeluga et al.,^(^ ^117^ ^)^ conducted a prospective randomized study comparing the use of parenteral nutrition with enteral nutrition in a patient undergoing HSCT, and concluded that parenteral nutrition was associated with greater use of diuretics, higher incidence of hyperglycemia and more catheter-related complications. Moreover, there was no difference in the hematopoietic recovery rate, length of hospital stay or survival, and the therapy costs increased two to three times.^(^ ^117^ ^)^

In another study comparing the use of parenteral nutrition *versus* enteral nutrition, once again the former was associated with major venous access-related complications^(^ ^72^ ^,^ ^118^ ^)^ and delayed platelet grafting after HSCT.^(^ ^119^ ^)^

Considering the controversies regarding the benefits of TPN, a nutritional screening protocol should be applied to identify the patients that would most benefit from this type of intervention. Some of the criteria suggested for the use of TPN would be severe malnutrition on admission, a long period of insufficient nutrient intake (approximately 7 to 10 days), and weight loss of more than 10% during the treatment.^(^ ^120^ ^)^

According to international recommendations,^(^ ^72^ ^,^ ^121^ ^)^ so far there is no age limit for the indication of TPN. However, it is consensual that TPN is indicated for patients who cannot have their nutritional requirements met by the oral/enteral route. TPN should be used in elderly patients in case of a fasting period of more than 3 days or if insufficient energy intake is expected for more than 7 to 10 days, and the enteral route is not possible or tolerated.^(^ ^113^ ^)^ Metabolic complications are more frequent in elderly people (*e.g.*, hyperglycemia, heart and kidney dysfunction), which warrants the use of high-lipid-content solutions.^(^ ^113^ ^)^ So far, no additional benefits have been seen with the addition of vitamins and antioxidants,^(^ ^56^ ^)^ except for reduction of bloodstream infection rates when glutamine is added to TPN.^(^ ^72^ ^,^ ^118^ ^)^

In short, during HSCT, TPN should be reserved for patients who cannot orally ingest at least 50% of their energy requirements and who are intolerant to enteral nutrition.^(^ ^115^ ^)^ Oral and enteral nutrition should always be preferred, and parenteral is indicated only in cases of gastrointestinal failure or intolerance to enteral/oral diet.

## MOST COMMON NUTRITIONAL COMPLICATIONS

The conditioning regimen for HSCT, including intensive chemotherapy and total body irradiation, may cause harmful effects on the digestive tract, such as mucositis, nausea, vomiting and diarrhea, resulting in inadequate oral intake and gastrointestinal malabsorption.^(^ ^122^ ^,^ ^123^ ^)^

Most patients start the treatment in an eutrophic state, with rapid depletion of their nutritional status by the direct toxic effects of treatment, or due to secondary complications, such as infections and GVHD. Chemotherapy-related side effects, such as nausea, vomiting, dysosmia, dysgeusia, xerostomia, sialorrehea, mucositis, hyporexia, diarrhea, constipation, abdominal discomfort and early satiety affect dietary intake.^(^ ^56^ ^,^ ^124^ ^)^ Also, the incidence of chemotherapy-induced toxicity is higher in malnourished patients, which can be due to an overestimation of the drug dose, usually calculated based on patient weight, rather than their body composition.^(^ ^56^ ^)^

The gastrointestinal tract is the organ most affected by the conditioning regimen due to its rapid division and regeneration.^(^ ^123^ ^)^ Conditioning induces an intense inflammatory response, which can damage the gastrointestinal tract with varying degrees of mucositis and, due to the loss of function of epithelial cells, increase the chance of bacterial translocation and abdominal infections.^(^ ^125^ ^)^

Painful ulcers and sores in the mouth, lips, gums and throat usually occur 5 to 7 days after conditioning, and can be seen in up to 90% of cases.^(^ ^125^ ^)^ Gastroesophageal symptoms include gastric reflux, esophageal dysmotility and gastric stasis secondary to excessive medication use, infections and local inflammation.^(^ ^126^ ^)^

Diarrhea occurs in more than half of patients due to high-dose chemotherapy, bacterial infections such as *Clostridium difficile*, use of antibiotics and GVHD.^(^ ^123^ ^)^ As a result of immunosuppression, some viral infections, such as herpes simplex, cytomegalovirus and varicella-zoster, as well as other viral enterites, such as rotavirus, norovirus and adenovirus, and intestinal parasite diseases (*Giardia lamblia* and cryptosporidiosis), may affect the entire gastrointestinal tract, causing symptoms.^(^ ^123^ ^)^ Another common complication of HSCT is GVHD.

## PALLIATIVE CARE IN HEMATOPOIETIC STEM CELL TRANSPLANTATION

According to the World Health Organization (WHO), palliative care (PC) is the approach to promote quality of life for patients and family members who are facing problems associated with life-threatening diseases, preventing and relieving their suffering through early identification, assessment and treatment of pain and other physical, psychosocial and spiritual problems.^(^ ^127^ ^,^ ^128^ ^)^

Incorporating PC to HSCT is still a challenge and a novelty to HSCT centers all over the world.^(^ ^129^ ^)^ Usually, the PC team is called upon only at the end of life of patients and/or after a long period in the intensive care unit.^(^ ^129^ ^)^ However, the implementation of an early PC program during HSCT, not only in the previously mentioned conditions, leads to improvements in quality of life, anxiety and depression indices, and in cost-effectiveness of the treatment, since it allows for better control of physical and emotional symptoms throughout the process.^(^ ^130^ ^,^ ^131^ ^)^ From the perspective of nutrition, some symptoms and complications, such as pain, mucositis, depression, nausea, loss of appetite and fatigue, which are so common during HSCT and directly affect the oral acceptance of patients, improve as soon as the PC team is involved.^(^ ^129^ ^)^

### Nutrition and palliative care

Eating behaviors and memory are associated to dietary beliefs and habits based on culture and family tradition, as well as to symbolisms of prosperity, health, strength, love, care and affection.^(^ ^128^ ^)^ Memories of taste, flavor, texture and smell of foods are associated with important activities and events.^(^ ^128^ ^)^ Therefore, food has a meaning that goes far beyond physiological needs.^(^ ^128^ ^)^

The goal of NT in PC could be increasing longevity in some situations, but must always be focused on promoting quality of life.^(^ ^132^ ^)^

For patients on PC outside the period considered as end of life, there is nothing different, nutritionally-speaking, from what has been discussed throughout this consensus. The main differences in the goals of nutritional care and approaches will occur at the end-of-life period.

## END OF LIFE

The end of life is defined as the phase during which the patient is living with a disease that will invariably lead to death.^(^ ^133^ ^)^ The earlier the approach, the better the control of symptoms, the psychological and social support offered to patients and family members, and the care-related decision-making in this phase of the disease.^(^ ^133^ ^)^

The role of PC at the end of life involves improving quality of life, taking into consideration the suffering, the preferences and values of the patient, caregivers and family, allowing for the best psychological, physical, religious and social support as possible, and grows in importance as death approaches.^(^ ^133^ ^-^ ^135^ ^)^ Some signs may help identify the last days of life, such as mottled skin, cold extremities, mouth breathing with a hyperextended neck, Cheyne-Stokes respiration, calling out to dead relatives and friends, talking about preparations for a long trip, and periods of sleepiness.^(^ ^134^ ^)^

In 2010, the Brazilian Medical Ethics Code endorsed end-of-life care aiming at a good death with dignity and without the need for useless therapies that do not bring any benefits to patients, which is known as orthothanasia.^(^ ^135^ ^)^ This term derives from the Greek words *orthós* (normal) and *thanatos* (death), and is defined as the natural process of death, without the use of therapeutic resources to postpone this outcome.^(^ ^135^ ^-^ ^137^ ^)^ In orthothanasia, death occurs as a result of abstaining from, abolishing or limiting treatments considered disproportionate to the terminality of the patient, without it being sought or triggered by the team or the patient.^(^ ^136^ ^)^

Another important term related to the end of life is dysthanasia, which in Greek means “dys” (detachment) and “thanatos” (death), which occurs when therapeutic measures which are not indicated and/or considered useless are taken, leading to unnecessary suffering of terminal patients.^(^ ^135^ ^-^ ^139^ ^)^ In dysthanasia, quality of life comes second, and therapeutic efforts becomes an obstinacy before the impossibility of a cure.^(^ ^138^ ^,^ ^139^ ^)^

Euthanasia is different from orthothanasia and dysthanasia, and can be translated as “good death”, “death without pain” or “death without suffering”.^(^ ^138^ ^)^ Euthanasia, which can be classified into active, passive, voluntary and involuntary, is not legally permitted in some countries, including Brazil.^(^ ^135^ ^-^ ^137^ ^)^ Active euthanasia is characterized by direct participation of the physician, who administers lethal drugs to cause death; passive refers to withdrawal or the non-introduction of therapeutic measures - in this case, measures are not considered useless, and their absence leads to death; voluntary means a request by the patient for an intervention that leads to death; and involuntary is when the patient does not consent to any active or passive practice that leads to death.^(^ ^135^ ^,^ ^136^ ^,^ ^138^ ^)^

The last concept is assisted suicide, characterized by death caused by the patient with the complicity and/or intentional help from another person, who can be a physician and/or a family member.^(^ ^136^ ^-^ ^139^ ^)^ This practice is also illegal in the Brazilian territory.

### Nutrition at the end of life

At the end of life, there is weight loss secondary to asthenia and cachexia, especially in cancer patients.^(^ ^140^ ^)^ Many symptoms, such as anorexia, xerostomia, nausea, dysgeusia, early satiety, dysphagia, weakness and confusion, can contribute directly or indirectly to decreased oral intake in these patients.^(^ ^140^ ^,^ ^141^ ^)^ Notwithstanding, most patients at the end of life show a significant reduction in appetite and thirst, expressing satiety with smaller quantities of food.^(^ ^142^ ^)^

The use of artificial feeding, whether by enteral or parenteral nutrition, has been proven to have no clinical benefit - on the contrary, it may even cause suffering to the patient.^(^ ^108^ ^,^ ^127^ ^,^ ^140^ ^,^ ^142^ ^,^ ^143^ ^)^

Enteral nutrition has been associated with deleterious side effects, such as pain, bleeding at the tube insertion site, diarrhea, constipation, aspiration, electrolyte deficiency, hyperglycemia and refeeding syndrome.^(^ ^140^ ^)^ Parenteral nutrition can cause more severe complications such as sepsis, fluctuating blood glucose, liver dysfunction, electrolytic changes and hypervolemia.^(^ ^140^ ^)^

The goal of NT should be focused on improving quality of life and promoting comfort. Therefore, individual needs should be respected, as well as patients’ dietary habits and preferences, control of symptoms and satisfaction.^(^ ^108^ ^,^ ^132^ ^,^ ^141^ ^,^ ^143^ ^,^ ^144^ ^)^

Considering all these items, for patients at the end of life, the nutritional approach should be the following: offer foods that bring comfort and satisfaction, consistent with the wishes of the patient; respect their refusal to eat, when present; offer half the portion, dividing foods in small quantities, if requested by the patient; wait the time they need to finish a meal, respecting their pace and preferred times of the day; do not restrict any foods (*e.g.*, do not restrict carbohydrates for a diabetic patient); do not give enteral and parenteral nutrition (only in exceptional cases); educate the multiprofessional team about the use of enteral and parenteral nutrition not bringing any benefit to the patient at the end of life; respect the patients’ wishes and autonomy; allow external foods that comply with hospital rules, in case of inpatients; improve the quality of life of the patient; and provide a peaceful and suitable place for patients to eat their meals.

Both PC and nutritional support must be present since the beginning of the HSCT procedure, to improve symptoms and the quality of life of patients and family members. Prioritizing comfort and respecting patients’ autonomy increasingly become the main focus of patient care when curative therapies fail, and the end of life draws nearer. In this setting, it is vital to understand that the meaning and symbolism of eating for each individual patient and their family have an importance that transcends the limits of physiological and nutritional requirements.
